# Identification of Adenovirus E1B-55K Interaction Partners through a Common Binding Motif

**DOI:** 10.3390/v15122356

**Published:** 2023-11-30

**Authors:** Nafiseh Chalabi Hagkarim, Wing-Hang Ip, Luca D. Bertzbach, Tareq Abualfaraj, Thomas Dobner, David P. Molloy, Grant S. Stewart, Roger J. Grand

**Affiliations:** 1 Institute for Cancer and Genomic Sciences, The Medical School, University of Birmingham, Birmingham B15 2TT, UK; 2 Leibniz Institute of Virology, Department of Viral Transformation, 20251 Hamburg, Germany; 3 Department of Medical Microbiology and Immunology, Taibah University, P.O. Box 344, Madinah 41477, Saudi Arabia; 4 Department of Biochemistry and Molecular Biology, School of Basic Medical Science, Chongqing Medical University, Chongqing 400016, China

**Keywords:** adenovirus, cullin, early region 1B, MRE11, p53, p53 binding motif, PR619, USP

## Abstract

The adenovirus C5 E1B-55K protein is crucial for viral replication and is expressed early during infection. It can interact with E4orf6 to form a complex that functions as a ubiquitin E3 ligase. This complex targets specific cellular proteins and marks them for ubiquitination and, predominantly, subsequent proteasomal degradation. E1B-55K interacts with various proteins, with p53 being the most extensively studied, although identifying binding sites has been challenging. To explain the diverse range of proteins associated with E1B-55K, we hypothesized that other binding partners might recognize the simple p53 binding motif (xWxxxPx). In silico analyses showed that many known E1B-55K binding proteins possess this amino acid sequence; therefore, we investigated whether other xWxxxPx-containing proteins also bind to E1B-55K. Our findings revealed that many cellular proteins, including ATR, CHK1, USP9, and USP34, co-immunoprecipitate with E1B-55K. During adenovirus infection, several well-characterized E1B-55K binding proteins and newly identified interactors, including CSB, CHK1, and USP9, are degraded in a cullin-dependent manner. Notably, certain binding proteins, such as ATR and USP34, remain undegraded during infection. Structural predictions indicate no conservation of structure around the proposed binding motif, suggesting that the interaction relies on the correct arrangement of tryptophan and proline residues.

## 1. Introduction

Human adenoviruses (HAdVs) constitute a large family of more than 100 different types, divided into seven species (A to G) [1]. Generally, they are responsible for relatively mild infections of the respiratory tract, the gastro-intestine, and the eye. However, in immunocompromised patients, such as those undergoing transplantation, adenoviruses pose a serious health risk, often resulting in high mortality rates [2,3].

Adenoviruses have a double-stranded linear DNA genome of about 35 kb, which is transcribed in both directions and encodes early, intermediate, and late proteins. Adenovirus early region 1 (E1) comprises two transcription units—E1A and E1B—and both are translated to give two major proteins [4,5]. The two E1A proteins, translated from 12S and 13S mRNAs, are identical over most of their sequence. Simplistically, through a multitude of interactions with cellular targets, they cause cell cycle progression into a ‘pseudo-S phase’, which facilitates the expression of other adenovirus genes, making use of the host cell transcription/translation machinery [4,6]. Adenovirus E1B proteins are transcribed from a 22S mRNA. The two major proteins, E1B-19K and -55K, are translated in different reading frames, so they have no sequence homology. E1B-19K has limited homology to Bcl2 and similarly adopts an anti-apoptotic role both during viral infection and in E1-transformed cells [5,7].

E1B-55K also appears to protect infected cells against apoptosis, for example, by causing the rapid degradation of p53 in the case of the species A and group C viruses or by direct interaction, resulting in the inhibition of transcriptional activity [8,9,10,11,12,13]. Adenovirus E1B-55K-mediated protein degradation is accomplished by association with E4orf6, recruitment of a cellular E3 ubiquitin ligase comprising a cullin (cullin 5 in the case of HAdV-C5 and Cul2 in the case of HAdV-A12), elongins B and C and RING Box 1 (Rbx1), ubiquitination of the substrate, and degradation by the proteasome [9,14,15]. E1B-55K serves as the substrate recognition component whereas E4orf6 binds to elongin C.

Group A (HAdV-A12, for example) and C (HAdV-C2 and HAdV-C5, for example) adenoviruses also target an appreciable number of other cellular proteins for degradation [14,16,17]. Reports over the last two decades have shown that many of these are associated directly, or indirectly, with the DNA damage response (DDR); for example, BLM, MRE11, DNA ligase IV, TNK1BP1, TOPBP1 and p53 have all been shown to associate with HAdV-C5 E1B-55K and, in most cases HAdV-A12 E1B-55K, and are subsequently degraded [14,18,19,20,21,22]. A number of cellular proteins involved in chromatin remodeling, such as Daxx, ATRX, and SPOC1, are also degraded in an E1B-55K-dependent manner [23,24,25]. It is interesting to note that there is no requirement for E4orf6 for the degradation of Daxx. The degradation of many other proteins not associated with the DDR or chromatin remodeling—for example, integrin α3, Fas, and EPHA7—has also been reported [26,27].

As well as determining the level of cellular proteins, HAdV-C5 E1B-55K also plays an important role in the preferential export of viral RNA from the nucleus and accumulation in the cytoplasm late in infection at the expense of host cell RNA [28,29]. In addition, this phenomenon is attributed to increased viral RNA levels during the later stages of infection, where they evidently outcompete the export of cellular RNA [30].

Notably, certain proteins undergo ubiquitination without being subjected to degradation. It has been shown that the RNA binding proteins, RALY and hnRNP-C, are targets for HAdV-C5 E1B-55K/E4orf6-mediated ubiquitination but not degradation, and this contributes to increased levels of viral RNA splicing and progeny production [27]. It is considered that E1B-55K has a role in the release of mRNA from viral replication centers, when the mRNAs can interact with the Nxf1/Tap export pathway [31,32]. Whether the interaction with RALY, hnRNP-C, or other RNA binding proteins associated with HAdV-C5 E1B-55K is involved in this process is not clear at present. The binding of E1B-55K to hnRNPUL1 (E1B-AP5) and hnRNPUL2 is likely also required for the localization of mRNAs [33].

It is generally accepted that knowledge of a protein’s secondary structure plays a crucial role in unraveling its mechanism of action. HAdV-C5 E1B-55K likely has a complex secondary and tertiary structure. Structural predictions have suggested that the N-terminal and C-terminal regions are largely random coils whereas the central region, from about amino acids 200 to 350, forms a hydrophobic core. This region has been modeled as a β-solenoid fold, based on the known structure of the snake adenovirus LH3 protein [34,35,36,37]. Although the sequence similarity between LH3 and E1B-55K is only slight, it does seem likely that much of the E1B-55K protein is highly structured since relatively minor substitutions, insertions, and/or deletions have major effects on its biological properties [38,39,40,41,42,43]. Considering these observations, it is not surprising that it has been particularly difficult to pinpoint binding sites on E1B-55K for the known cellular interactors. It has been suggested that the binding site for p53 is towards the central region of HAdV-C2/5 E1B-55K (amino acids 224–354) although most studies fail to map this precisely; for example, deletion or insertion mutants at H^180^, R^240^, H^260^, A^262^, R^309^, and H^326^ disrupt the interaction [33,39,41,42,44,45]. However, a binding site for an additional E1B-55K binding protein, USP7, was localized to the N-terminal 71 residues of HAdV-C5 E1B-55K [20].

While binding domains/interaction regions on cellular proteins have been identified [46,47], exact binding sites for E1B-55K on its target proteins have generally not been mapped, apart from USP7 and p53 [20,48]. A detailed investigation of the binding site on p53 for HAdV-C5 E1B-55K was performed using a series of proteins with point mutations. A short hydrophobic region close to the N-terminus of p53 has been shown to comprise the HAdV-C5 E1B-55K binding site. with W^23^ and P^27^ considered to be of particular importance [48]. There is evidence suggesting the participation of a comparable region in the interaction between p53 and MDM2 [48]. In view of the multiplicity and diversity of the reported HAdV-C5 E1B-55K binding proteins (summarized in [34], but also see [27]) and the observation that some of the proposed interactions have only limited effect on viral replication [19,21,26], we considered the possibility that an identical motif could be present in some of the binding proteins and the interactions might be fortuitous. In this manuscript, we have identified novel HAdV-C5 E1B-55K binding partners based on the presence of the p53 xWxxxPx binding motif [48]. A number of these have also been shown to be degraded during adenovirus infection in a cullin-dependent manner. Recognition by HAdV-C5 E1B-55K appears to be largely if not entirely through the correctly spaced tryptophan and proline residues in the xWxxxPx sequence. It is important to note, however, that other sequences in the E1B-55K binding proteins could be involved in the interactions. In this study, our focus is specifically on substrates published for HAdV-C5 and -A12, unless stated otherwise. However, it is important to acknowledge that substrates for additional HAdVs have also been identified.

## 2. Materials and Methods

### 2.1. Cell Lines, Virus Infection and DNA Transfection

HeLa and U2OS cells were obtained from ATCC. HAdV-C5 E1-expressing HEK293 cells [49] were a generous gift from Frank Graham. NBS1-negative U2OS cells [50] were a generous gift from Manuel Stucki (University of Zurich). HAdV-A12 E1HER2 (HER2) cells have been described previously [51]. All cells were grown in Dulbecco’s modified Eagle’s medium (DMEM) (Sigma-Aldrich, St. Louis, MO, USA) supplemented with 8% fetal calf serum. Cells were infected with wt HAdV-C5 at an infectivity of 5 plaque-forming units (pfu) per cell, HAdV-C5 dl1520 [52] and H5pm4155 [19] with 10 pfu per cell, or HAdV-A12 dl703 [53] at an infectivity of 20 pfu per cell. In some experiments, 5 µM MLN4924 (MedChemExpress, Monmouth Junction, NJ, USA) was added to cells during viral infection to inhibit cullin activity, as described [19], or 6 µM or 20 µM PR619 (Tocris, Bristol, UK) was added during viral infection to inhibit USP activity. In all cases, cells were pre-incubated with the appropriate drug for 1 h before viral infection. HeLa cells were transfected using Lipofectamine 2000 (Invitrogen, Waltham, MA, USA), with constructs expressing N-terminally HA-tagged E1B-55K from HAdV-E4, D9, B34, and F40, and incubated for 48 h before harvesting.

### 2.2. SDS-PAGE, Immunoblotting and Antibodies

Infected cells were harvested in ice-cold PBS, and proteins were solubilized in 9 M urea, 40 mM Tris HCl pH 7.4, and 0.15 M β-mercaptoethanol. Prior to immunoblotting, lysates were fractionated on 10% or 8% SDS polyacrylamide gels. Proteins were electrophoretically transferred to nitrocellulose membranes before incubation with antibodies at 4 °C overnight. The following antibodies were used in this study: HAdV-C5 E1A, M73 [54]; HAdV-C5 E1B-55K, 2A6 [55] or a rabbit antibody raised against GST-HAdV-C5 E1B-55K (produced in house); and p53 (DO-1; generous gift from David Lane). Commercial antibodies were obtained as follows: SQSTM1 (p62) (Abcam, Cambridge, UK); ATR, CSB, LETM1, MRE11, NBS1, TNKS 1 and 2, USP6, USP7, USP9, USP15, USP33, and USP34 (all from Santa Cruz Biotechnology, Dallas, TX, USA); and BLM, DNA ligase IV, and DNMT1 (all from Bethyl Laboratories, Montgomery, TX, USA).

### 2.3. Immunoprecipitation and Peptide Pull-Down Assays

Cells were solubilized in NETN buffer (0.15 M NaCl, 40 mM Tris HCl pH 7.4, 5 mM EDTA, and 1% NP40) and clarified by centrifugation at 40,000× *g* for 30 min. Lysates were then incubated at 4 °C overnight with appropriate antibodies (co-immunoprecipitation). We used the 2A6 E1B-55K antibody or the rabbit antibody produced in-house for co-immunoprecipitation experiments, as appropriate. Antigen–antibody conjugates were captured with protein G-agarose beads (2 h incubation). After washing with NETN buffer, protein complexes were released in the SDS sample buffer. A synthetic peptide identical to an N-terminal region of human p53 (^17^ETFSDLWKLLPENNVLS^33^-Ahx-K-(Biotin)-amide) or a ‘mutated’ form (^17^ETFSDLAKLLAENNVLS^33^-Ahx-K-(Biotin)-amide) linked to biotin was used for pull-down assays. In this case, HEK293 and HER2 cell lysates and lysates prepared from HeLa cells transfected with HA-E1B-55K constructs from other adenovirus species, prepared as described, were incubated with peptide for 3 h. Peptides were captured using streptavidin-agarose beads (1 h incubation) and then released, together with bound proteins, for electrophoresis with an SDS sample buffer.

### 2.4. Immunofluorescence Microscopy

Microscopy was performed essentially as described previously [19]. Briefly, HeLa cells were grown on glass coverslips and then infected with HAdV-C5 (5 pfu per cell). After 24 h, cells were fixed in 3.6% paraformaldehyde for 10 min and then extracted in 0.5% Triton X-100 in PBS for 5 min. Fixed cells were stained with primary antibodies overnight at 4 °C, washed three times in PBS, and stained with secondary antibodies for 1 h. DNA was stained with 4′,6-diamidino-2-phenylindole (DAPI). Fluorescence images were taken by using a Nikon E600 Eclipse 333 microscope equipped with a 60× oil lens, and images were acquired and analyzed by using Volocity software 334 v4.1 (Improvision, Mountain View, CA, USA). Antibodies used were the same as those listed for Western blotting.

### 2.5. Structural Predictions

Sequences for all xWxxxPx-containing candidate proteins were obtained from NCBI (http://ncbi.nlm.nih.gov accessed between 5 February 2021 and 3 June 2022) and UniProtKB (https://www.uniprot.org accessed between 5 February 2021 and 3 June 2022). Amino acid sequence alignments were derived from comparisons between the MUSCLE and Clustal Omega (https://www.ebi.ac.uk accessed between 5 February 2021 and 3 June 2022 [56]) algorithms with MAFFT (https://mafft.cbrc.jp accessed between 5 February 2021 and 3 June 2022 [57]), set to default parameters and constrained to the input reference sequence for human TP53 protein (UniProtKB accession number P04637). Protein logos were computed for xWxxxPx motifs using Weblogo3 (http://weblogo.threeplusone.com accessed between 5 February 2021 and 3 June 2022 [58]) referenced to a full scale of 4.3 bits of information within the full-length p53 protein. Structure predictions for proteins containing xWxxxPx-motifs were derived for short sequences (23 amino acids) from within each protein, commencing between 1 and 19 amino acids for the N-terminal (NBS1 and ERCC61, respectively) and terminating between 3 and 21 residues for the C-terminal (ERCC6 and NBS1), for the motif in each instance using PSI-PRED (bioinf.cs.ucl.ac.uk/psipred; accessed between 5 February 2021 and 3 June 2022 [59]), Jpred4 (www.compbio.dundee.ac.uk/jpred accessed between 5 February 2021 and 3 June 2022 [60]), and PREDATOR (https://npsa-prabi.ibcp.fr accessed between 5 February 2021 and 3 June 2022 [61]) fold recognition algorithms. From these, weighted averages of structural preference for each amino acid within every sequence were generated.

## 3. Results

As mentioned, the binding site on p53 for the HAdV-C5 E1B-55K protein was closely mapped to a short N-terminal hydrophobic region between amino acids 23 and 27, with the tryptophan (W; amino acid 23) and proline (P; amino acid 27) residues considered to be of particular importance [48]. Because of the multiplicity and diversity of the identified E1B-55K binding proteins, we reasoned that this might be explained by the recognition, by the viral protein, of a comparable sequence (xWxxxPx) present in cellular target proteins. Some support for this idea comes from the most extensive study of E1B-55K co-immunoprecipitating proteins in which an appreciable proportion (37%) of those identified contained the amino acid sequence (Table 1, groups I and II) [62]. Similarly, in a study to identify cellular proteins ubiquitinated in the presence of HAdV-C5 E1B-55K and HAdV-C5 E4orf6 that were subsequently degraded, 44% contained an xWxxxPx motif [27] (Table 1, group IV).

### 3.1. Identification of Novel HAdV-C5 E1B-55K Binding Proteins

We first looked to see if other proteins, containing the xWxxxPx sequence, would also bind to HAdV-C5 E1B-55K. The nine candidates examined in this study (ATR, CHK1, CSB (ERCC6), DNMT1, LETM1 tankyrase (TNKS), USP9, USP33, and XPF) tended to be proteins primarily, although not exclusively, related to the DDR, but they were otherwise chosen at random.

**Figure 1:**
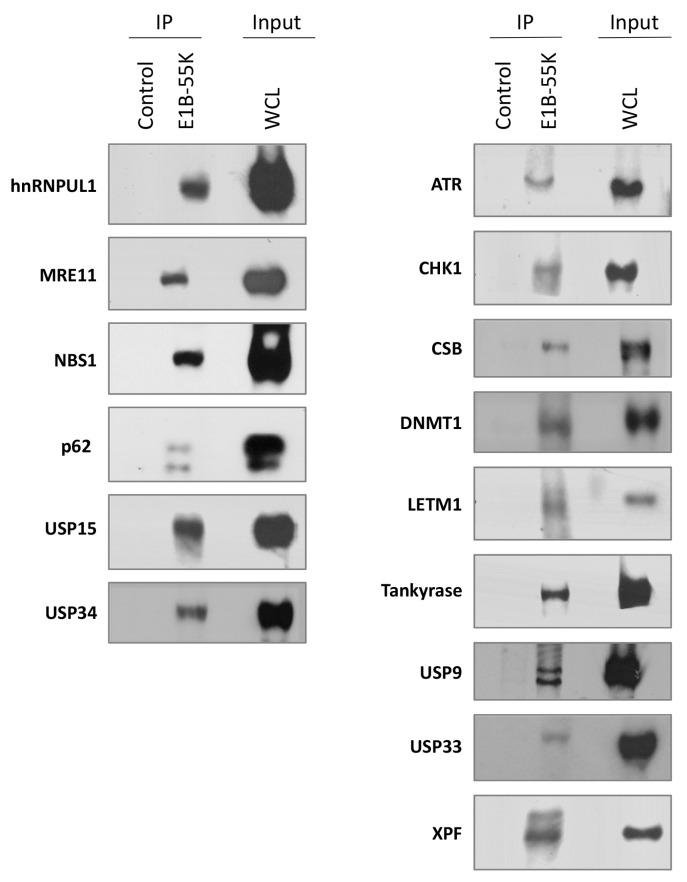
The interaction of HAdV-C5 E1B-55K with novel cellular binding partners. Lysates from HEK293 cells were immunoprecipitated with antibodies (either mouse or rabbit as appropriate) against the HAdV-C5 E1B-55K protein. After immunoblotting, interacting proteins were detected with the antibodies shown. Known binding proteins such as NBS1, MRE11, and hnRNPUL1 are included for comparison (left column). “Control” is an irrelevant antibody included as a negative control, raised against either collagen type IV (rabbit) or vimentin (mouse). Images represent the results of three repeated experiments. WCL, whole cell lysate.

Proteins were immunoprecipitated from HEK293 cells using either mouse or rabbit antibodies against HAdV-C5 E1B-55K and immunoblotted for the potential binding partner (Figure 1). It can be seen that ATR, CHK1, CSB (ERCC6), DNMT1, LETM1, TNKS 1 and/or 2 (the antibody used recognized both TNKS 1 and 2), USP9, USP33, and XPF all co-immunoprecipitated with the viral protein. Co-immunoprecipitations of the well-characterized binding partners hnRNPUL1, MRE11, and NBS1 are included for comparison, as well as SQSTM1 (p62), USP15, and USP34, which had previously been identified in a mass spectrometry screen [62]. Confirmatory co-immunoprecipitations, carried out with specific antibodies and immunoblotted for HAdV-C5 E1B-55K, are shown in Figure S1. The amino acid sequences around the potential binding sites of all the interacting proteins are shown in Table 1, and those identified in this study are in Table 1, group VI.

### 3.2. Interaction of Adenovirus E1B-55K Proteins with the xWxxxPx Sequence

**Figure 2:**
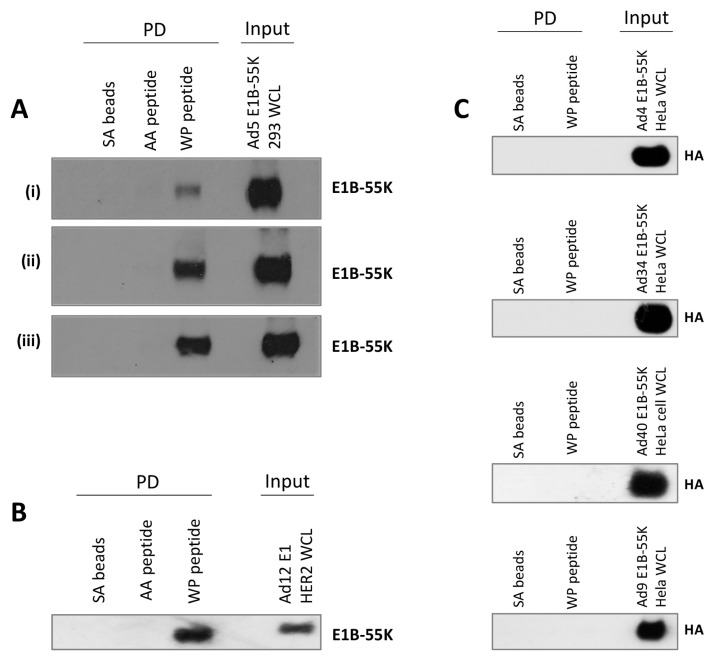
The interaction of adenovirus E1B-55K proteins with the xWxxxPx motif. (**A**) HEK293 cell lysates were mock incubated or incubated with either biotin-linked ETFSDLWKLLPENNVLS peptide (WP peptide) or ETFSDLAKLLAENNVLS peptide (AA peptide), (i) 1 µg/mL, (ii) 5 µg/mL and (iii) 20 µg/mL for 2 h. Streptavidin (SA) beads were added for a further 90 min. After washing bound proteins were released with SDS sample buffer and fractionated by PAGE. Bound HAdV-C5 E1B-55K was detected by immunoblotting. (**B**) HER2 cell lysate was incubated with WP peptide or AA peptide (10 µg/mL) as in (**A**). Bound HAdV-A12 E1B-55K was detected by immunoblotting. (**C**) HeLa cells were transfected with pcDNA3 constructs expressing HA-tagged E1B-55K proteins from Ads 4, 34, 40, and 9. After 48 h, cell lysates were incubated or mock incubated with WP peptide (10 µg/mL) for 2 h. After incubation with streptavidin beads samples were processed as in (**A**) and then immunoblotted for the HA tag. Images represent the results of three repeated experiments. WCL, whole cell lysate.

To confirm that the HAdV-C5 E1B-55K interaction only requires a short sequence encompassing the xWxxxPx sequence, a lysate from HEK293 cells was incubated with a biotin-linked peptide identical to the N-terminal sequence of p53 (^17^ETFSDLWKLLPENNVLS^33^). After a pull-down with streptavidin beads, interacting proteins were fractionated by SDS-PAGE and HAdV-C5 E1B-55K identified by immunoblotting (Figure 2A). Pull-down with a similar peptide in which the tryptophan and proline residues were substituted with alanines (^17^ETFSDLAKLLAENNVLS^33^) showed no interaction (Figure 2A). Increasing amounts of peptide resulted in increased ‘pulled down’ HAdV-C5 E1B-55K (Figure 2A). In a similar experiment, the two peptides were incubated with lysates from HER2 cells. HAdV-A12 E1B-55K bound to the ETFSDLWKLLPENNVLS peptide but not to that containing alanines (Figure 2B).

To determine whether the same p53 amino acid sequence was recognized by E1B-55K proteins from other HAdV species, plasmids encoding HA-tagged versions of HAdV-E4 (species E), HAdV-D9 (species D), HAdV-B34 (species B) and HAdV-F40 (species F) E1B-55K proteins were transfected into HeLa cells. After 48 h, cell lysates were incubated with the biotin-tagged ETFSDLWKLLPENNVLS peptide. Interacting proteins were isolated using streptavidin beads and subjected to immunoblotting with an anti-HA antibody (Figure 2C). No interaction could be seen with the HAdV-E4, HAdV-D9, HAdV-B34, or HAdV-F40 E1B proteins, suggesting that the binding through xWxxxPx might differ between different HAdV species. This was somewhat unexpected as it has been previously reported by us and others that p53 binds to E1B-55K proteins from other HAdVs and in some cases is stabilized [8,12,67]. It should be noted, however, that these experiments are not directly comparable to those presented in Figure 1 as conditions are significantly different.

### 3.3. Adenovirus-Mediated Degradation of Novel Cellular Targets

**Figure 3:**
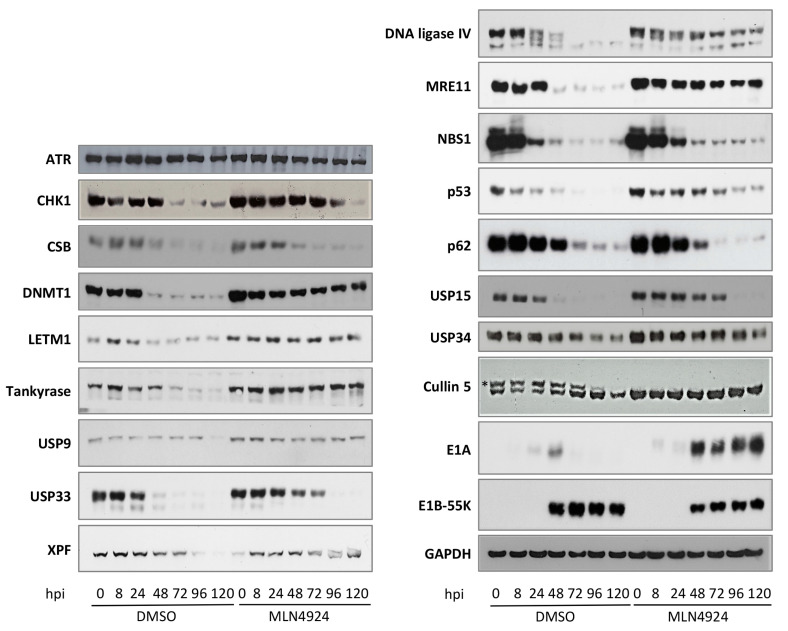
Degradation of novel binding partners during HAdV-C5 infection is dependent on cullin function. The **left**-hand blots display novel E1B-55K binding partners, while the **right**-hand blots serve as controls. HeLa cells were infected with HAdV-C5 at an infectivity of 4 pfu/cell, either in the presence or absence of 5 mM MLN4924. Cells were harvested at the times shown, post-infection, fractionated by PAGE, and immunoblotted with the antibodies as indicated. For the cullin 5 blots, * indicates the NEDDylated form. Images represent the results of three repeated experiments.

To date, the major consequence of HAdV-C5 E1B-55K binding during viral infection has been shown to be rapid proteasome-mediated protein degradation [9,16]. With this in mind, the expression of the proteins (shown in Figure 1 and Figure S1 to bind HAdV-C5 E1B-55K) was assessed over an extended time course of HAdV-C5 infection. A similar set of infections was carried out in the presence of the NEDD8 (NAE)/cullin inhibitor, MLN4924. It can be seen from Figure 3 that CHK1, CSB, DNMT1, LETM1, SQSTM1 (p62), TNKS, USP15, USP33, and XPF are all degraded during HAdV-C5 infection (Figure 3). Well-characterized HAdV-C5 targets such as DNA ligase IV, p53, MRE11, or NBS1 are included for comparison (Figure 3). The rates of protein degradation vary, with some proteins being very rapidly degraded (for example, p53, DNA ligase IV, USP33, and CSB), whereas others are more stable, such as SQSTM1 (p62) and TNKS. We have previously noted that TAB182 (TNKS1BP1) is also degraded relatively slowly during HAdV infection [19]. It is interesting to mention that some HAdV-C5 E1B-55K binding proteins containing the xWxxxPx sequence are not degraded at all during HAdV-C5 infection (Figure 3). Thus, there appears to be little or no reduction in the level of USP9, USP34, or ATR. This is consistent with observations of Herrmann and colleagues [27], who found that many proteins ubiquitinated by HAdV-C5 E1B-55K/E4orf6 were also stable. To confirm that the reduction in protein expression was due to cullin-based E3 ligase activity, viral infections were carried out in the presence and absence of MLN4924. MLN4924 is an inhibitor of cullin NEDDylation and has been shown to inhibit protein degradation during adenovirus infection [9].

It can be seen from Figure 3 that HAdV-induced protein degradation is generally reduced in the presence of the inhibitor, although it is not completely negated (for example, TNKS, LETM1, USP15, and USP33). In the case of CSB, NBS1, and SQSTM1 (p62), however, it appears that the inhibition of the cullins does not result in protein stabilization; whether this indicates degradation by a different pathway is unclear at present. The immunoblot of cullin 5 shows that the higher-molecular-weight NEDDylated (active) band is absent in the cells treated with MLN4924 (Figure 3). At the latest times in the experiment, there is a marked reduction in the proportion of the NEDDylated cullin 5. The reasons for this are not clear, but there appears to be sufficient active protein present to continue with the degradation. Alternatively, it is possible that substrates could have been ubiquitinated by about 80 hpi and subsequently degraded, when there was no further need for an active cullin.

In the experiments shown in Figure 3, no account has been taken of the possibility that host cell shut-off could have contributed to the reduction in the level of particular proteins examined. However, the fact that proteins were generally stabilized by MLN4924 suggests that this was not a significant factor but cannot be discounted.

### 3.4. Interaction of MRE11 with HAdV-C5 E1B-55K

**Figure 4:**
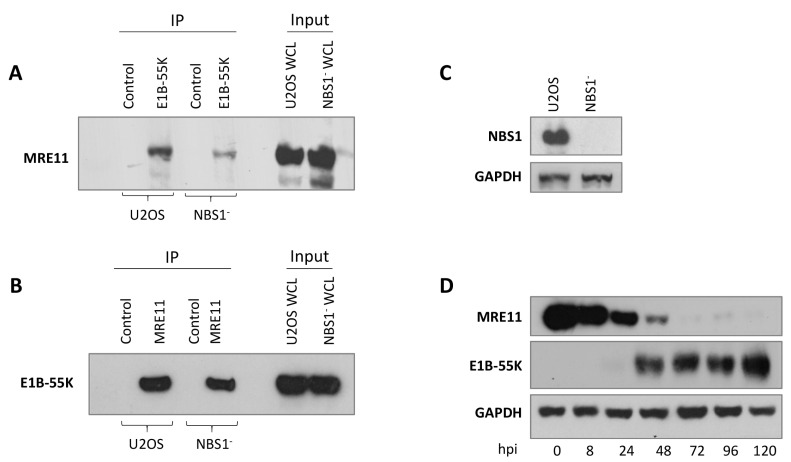
HAdV-C5 E1B-55K binds to MRE11 in the absence of NBS1. (**A**,**B**) U2OS or NBS1-negative cells were infected with H5pm4155 (E4orf3^−^, E4orf6^−^) at 10 pfu/cell for 48 h. Cells were harvested and immunoprecipitated with antibodies against either HAdV-C5 E1B-55K (**A**) or MRE11 (**B**). After PAGE and immunoblotting, co-immunoprecipitated MRE11 (**A**) or HAdV-C5 E1B-55K (**B**) was detected. Control, immunoprecipitated with an irrelevant antibody as in Figure 1. (**C**) Immunoblot showing no expression of NBS1 in NBS1^−^ cells. (**D**) NBS1^−^ cells were infected with HAdV-C5 (5 pfu/cell) and harvested at the times shown. Lysates were immunoblotted for MRE11, HAdV-C5 E1B-55K, and GAPDH as a loading control. Images represent the results of three repeated experiments. WCL, whole cell lysate.

It is clear from several studies that proteins that do not contain the xWxxxPx motif interact with HAdV-C5 E1B-55K and are degraded during adenovirus infection (for example, [25,27,62]). Notably, MRE11 does not have the sequence but NBS1 does (MW^2^KLLP^6^AAGP). We considered the possibility that NBS1 is the principal target for HAdV-C5, and MRE11 associates with E1B-55K as an integral component of the MRN complex. The reduction in the level of MRE11 could then be attributed to the instability of the complex resulting from the degradation of NBS1. To address this possibility, U2OS cells that do not express NBS1 [50] were infected with H5pm4155 (HAdV-C5 E4orf3^−^E4orf6^−^), which expresses E1B-55K but does not lead to the degradation of cellular targets [19]. MRE11 is expressed to similar levels in the NBS1^−^ and U2OS lines and is co-immunoprecipitated with E1B-55K (Figure 4A). In the complementary experiment, E1B-55K was immunoprecipitated with an MRE11 antibody (Figure 4B). The blot in Figure 4C illustrates the absence of NBS1 in the U2OS NBS1^−^ cell line. MRE11 is degraded as expected following the infection of NBS1^−^ cells with wild-type (wt) HAdV-C5 (Figure 4D). It is clear from these observations that MRE11 is a target for HAdV-C5 E1B-55K and interacts with it directly, with no requirement for the xWxxxPx motif.

### 3.5. Sub-Cellular Localization of Novel Adenovirus E1B-55K Binding Proteins during Infection

E1B-55K localizes to viral replication centers (VRCs) during infection. An appreciable number of DNA repair proteins have been also shown to localize to VRCs in adenovirus-infected cells, possibly relocalized by E1B-55K (for example, [68,69]; reviewed in [70]). We considered the possibility that proteins identified as novel interactors in Figure 1 and Figure S1 could also be present in VRCs. Therefore, we infected HeLa cells for 24 h with HAdV-C5. We fixed, permeabilized, and incubated the cells with antibodies for 18 h. The VRCs were visualized by staining with antibodies against the HAdV-C5 DNA binding protein (DBP) or RPA32, which has previously been shown to localize to VRCs [68]. The location of most of the cellular E1B-55K binding proteins examined was unaffected by HAdV infection (Figures S2–S4). Thus, CSB, DNMT1, LETM1, TNKS, USP9, and USP15 staining was very similar in the mock and infected cells, being pan-nuclear and/or cytoplasmic (Figures S2–S4). However, USP34 is clearly localized to VRCs. CHK1 staining was pan-cellular in uninfected cells, although it appeared to be predominantly cytoplasmic. Following infection, however, it was mainly observed in the nucleus, although not in VRCs. ATR was nuclear in uninfected cells but tended to be pan-cellular after HAdV-C5 infection; it also formed foci, although these did not correspond to VRCs (Figures S2–S4). 

### 3.6. Effect of Deubiquitinase (DUB) Inhibition on HAdV-C5 Infection

**Figure 5:**
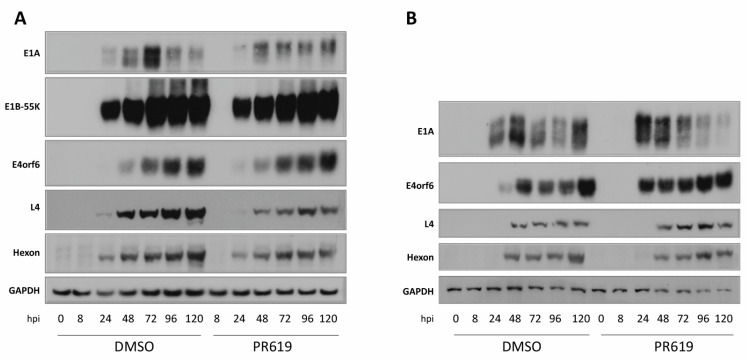
The effect of the DUB inhibitor PR619 on adenovirus infection. HeLa cells were treated for 1 h with 20 µM PR619 and infected with (**A**) HAdV-C5 (5 pfu/cell) or (**B**) *dl*1520 (10 pfu/cell). Cultures were then incubated in the presence of 20 µM PR619 and harvested at the times shown. Lysates were subjected to immunoblotting as shown. Images represent the results of three repeated experiments. L4, L4-100K.

Several proteins containing the xWxxxPx motif and interacting with HAdV-C5 E1B-55K are USPs and USP7 has been shown to bind to and interact with E1B-55K, although it does not contain the xWxxxPx motif [20]. Indeed, about half of the USPs contain the xWxxxPx sequence. In an overall study such as this, it is not feasible to deplete each USP in turn to determine which, if any, affects viral replication. Therefore, cells were treated with the non-selective broad-range inhibitor of deubiquitinating enzymes (DUBs), PR619 (for example, [71,72]), and then infected with HAdV-C5 or the E1B-55K-negative virus, *dl*1520. HeLa cells were pre-incubated with 20 µM PR619 for 1 h, infected with the virus for 2 h, and then incubated with medium containing the drug. Immunoblotting of an HAdV-C5-infected time course, in the presence and absence of 20 µM PR619, showed only minor differences in early viral protein expression, although E1A was at a higher level at early times in the absence of PR619 (Figure 5A).

Hexon levels were somewhat reduced in the presence of PR619, but the effect on L4-100K was much more marked, with appreciably reduced levels. The reasons for this are not clear at present. To determine whether the inhibition of DUBs is likely to affect adenovirus replication per se, infection was carried out in the absence of E1B-55K using the *dl*1520 mutant virus (Figure 5B). This removes any effects of interaction and/or USP degradation attributable to E1B-55K and looks at the effects of the DUB in isolation. However, again, differences in the presence of PR619 were not marked, although in this case there was no significant effect on L4-100K expression (Figure 5B).

### 3.7. Structural Prediction for the xWxxxPx Motif

**Figure 6:**
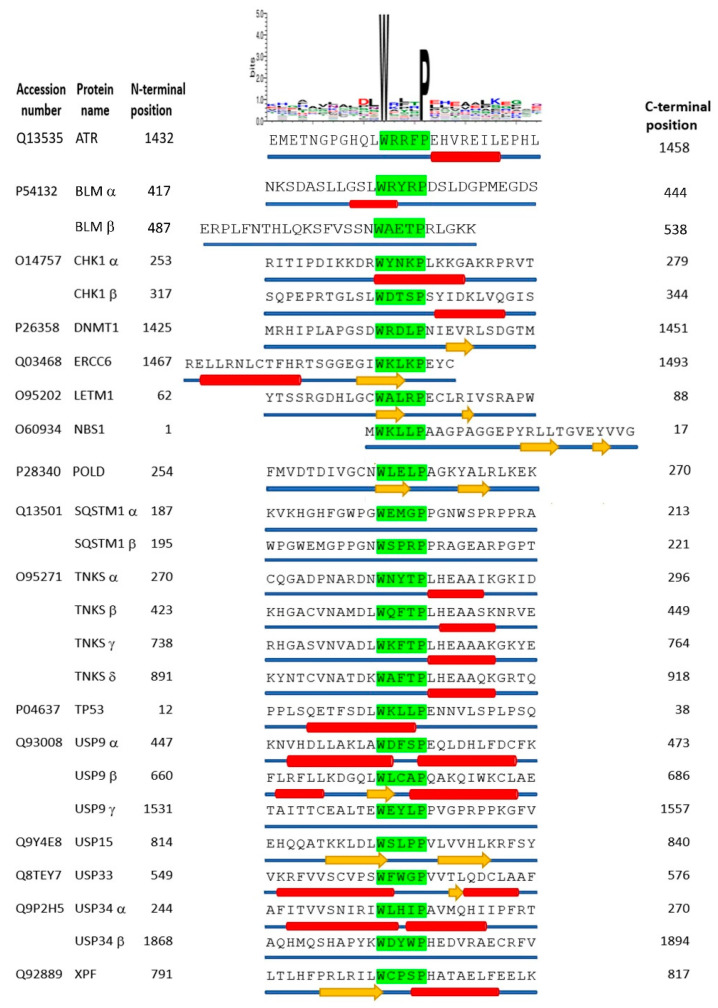
Structural prediction of xWxxxPx-containing motifs. The accession numbers of candidate proteins were obtained from the UniProtKB databank. Proteins containing xWxxxPx motifs (green background highlighted typeset) are listed alphabetically. The N- and C-terminal positions of each protein primary sequence are shown. Where a protein contains more than one xWxxxPx motif they are designated by Greek letters (α, β, γ, etc.). A weblogo cartoon of amino acids in the sequence of the proteins listed is shown at the top of the figure. Average predicted structural propensities for each amino acid in each protein are illustrated as either α-helix (red cylinder), β-strand (yellow arrow), or random coil (thin blue cylinder) below the primary sequence.

It is clear that the proteins identified as E1B-55K binding proteins do not have a conserved amino acid sequence except for the tryptophan and proline residues spaced three amino acids apart, except for a slight preponderance of hydrophobic amino acids (Table 1 and Figure 6).

We considered the possibility that the binding site could have a conserved secondary structure, however. Using the programs listed in the Materials and Methods section, structural predictions were carried out on a 27 amino acid region encompassing the xWxxxPx motif in the binding proteins shown in Figure 1. It can be seen that there is no conservation of predicted structure; some regions are unstructured (for example SQSTM1 (p62)) and some have adjacent α-helices either N-terminal to the tryptophan (p53), C-terminal to the tryptophan (TNKS1) or both upstream and downstream of the binding site (USP34 and USP9). The site in other proteins contains β-strands, such as in USP15 and LETM1 (Figure 6). We conclude that the two amino acids, W and P, are the sole determining factors for interaction with HAdV-C5 E1B-55K.

## 4. Discussion

The adenovirus early region 1 proteins exert their effect on target cells, both during infection and cellular transformation, through a complex series of protein–protein interactions, as neither E1A nor E1B-55K have enzymatic activity nor bind to DNA. A large number of interacting proteins have been identified for both early region proteins from HAdV-C5 and, to a lesser extent, from HAdV-A12 [5,6,34,73,74]. Whilst all the sites of interaction have been closely mapped on E1A, they remain largely elusive for E1B-55K. This seems to be because E1A is highly modular and contains distinct regions that exist in a dynamic, conformationally unstructured state, giving rise to interactions with short discrete amino acid sequences [6,74]. In this way, it has been suggested that adenovirus E1A forms a molecular hub with dozens of primary interactions and hundreds and possibly thousands of secondary interactions [6,74]. HAdV-C5 E1B-55K, on the other hand, probably has a complex secondary structure. This suggestion is based on the observations that certain point mutations and small deletions destabilize the HAdV-C5 E1B-55K protein; similarly, point mutations at disparate sites, particularly in the central region, interfere with substrate binding and other phenotypes [33,38,39,40,41,42,45]. Several laboratories have attempted the large-scale purification of E1B-55K, and, to our current understanding, none were successful. It is the complex secondary structure of the protein that appears to pose significant challenges in the purification process. Interestingly, the LH3 protein purified from a snake adenovirus, which has been considered to have functional similarities to E1B-55K, has been shown to be highly structured [35]. The modeling of HAdV-C5 E1B-55K suggests unstructured N- and C-terminal regions with a hydrophobic core [34,35,36,37]. Probably because of this structural integrity, binding sites for partner proteins have been difficult to pinpoint. In complementary studies, relatively little progress has been made in the determination of binding sites for E1B-55K on target proteins except for p53 and USP7 [20,48].

By virtue of the wide disparity in the identified E1B-55K interacting proteins and the observations that some, such as integrin α3, BLM, and TAB182 [19,21,26], appear to have relatively little effect on viral replication, we considered the possibility that these diverse interactions were due to the chance presence of a conserved motif rather than direct, ‘intentional,’ targeting by the virus. As far as we are aware, the only binding site for which detailed information is available is that on p53, where a short hydrophobic sequence has been identified [48], and within that sequence, the tryptophan and proline residues, separated by three amino acids, are of particular importance. The sequence is common in previously identified HAdV-C5 E1B-55K binding proteins (for example, DNA ligase IV, BLM, TAB182, and NBS1); nevertheless, it is certainly not essential for interaction (Table 1). Notably, it is absent from MRE11 and Daxx and many of the binding proteins identified in a mass spectroscopy screen by Hung and Flint [62]; it is also absent from many of the ubiquitinated proteins identified by Herrmann and colleagues [27] in their HAdV-C5 E1B-55K/E4orf6 screen. However, it occurred frequently enough to encourage us to investigate whether other xWxxxPx motif-containing proteins would also bind to HAdV-C5 E1B-55K. In fact, this sequence is relatively common in the mammalian proteome, such that it is unlikely to be the only determining factor for the interaction between HAdV-E1B-55K and its cellular targets. We chose to examine nine novel proteins picked largely at random but predominantly on the basis that they are involved in the DDR. HAdV-C5 E1B-55K co-immunoprecipitates from HEK293 cells with the proteins listed in Table 1 (group VI) and with three other proteins (USP15, USP34, and SQSTM1 (p62)), previously identified by Hung and Flint [62], which had not been characterized in the context of adenovirus biology. Obviously, our study represents only a very small proportion of proteins containing xWxxxPx but we did not encounter any that could not be immunoprecipitated with HAdV-C5 E1B-55K. Notably, both RALY and hnRNP-C lack the xWxxxPx motif. It is tempting to speculate that the interaction between these proteins and E1B-55K occurs through RNA rather than the “xWxxxPx” motif, considering that RALY, hnRNP-C, and E1B-55K are all recognized as RNA-binding proteins [37].

It has been observed that interaction with HAdV-C5 E1B-55K/E4orf6 during viral infection would mostly result in proteasome-mediated degradation, as has been observed for p53, DNA ligase IV, and BLM. We, therefore, investigated whether the novel HAdV-C5 E1B-55K interactors were also targeted to the proteasome. Most were degraded, although at varying rates (Figure 3)—for example, CSB and USP33 were degraded at a similar rate to p53 and MRE11, whereas TNKS and LETM1 were much more stable (Figure 3). Interestingly, however, ATR, USP9, and USP34 were largely unaffected over a prolonged time course of infection. The E1B-55K/E4orf6 complex may regulate its targets in a highly diverse manner. This regulation does not seem to be exclusively dependent on uniform degradation pathways for all targets; instead, it suggests the presence of a nuanced fine-tuning process. It would be interesting to examine whether these proteins were ubiquitinated (without degradation), as has been reported for an appreciable number of other proteins that are not degraded in the presence of HAdV-C5 E1B-55K/E4orf6 [27]. Considering the known interaction between cullin-5 and E1B-55K, however, it is probable that the viral protein may disrupt host cullin-RING ubiquitin ligases, a phenomenon observed in many other viral infections as well [62,75]. Remarkably, USP9 and USP34 deviate from the norm with three and two xWxxxPx motifs, respectively, distinguishing them from the majority of other E1B-55K targets. It is noteworthy that both proteins harbor D (aspartic acid) or L (leucine) in close proximity to W (tryptophan). This is particularly significant since the presence of a double/triple xWL/DxxPx motif may induce a conformation in E1B-55K that hinders its interaction with E4orf6. Moreover, the identification of a unique xWRRFPx motif in ATR (and in CEP170) raises intriguing questions. The striking presence of this distinct “arginine-rich” motif suggests that it may induce a conformational state in E1B-55K, potentially impeding its binding to E4orf6. This could be a plausible explanation for the observed resistance of ATR to degradation despite its interaction with E1B-55K. Future research will elucidate the functional implications of these interactions. Numerous studies have now shown the presence of large numbers of DNA repair proteins, as well as proteins involved in the synthesis of viral DNA and RNA, at adenovirus replication centers [12,68,76,77,78]. Only USP34 out of the E1B-55K binding proteins identified here localized to VRCs. USP34 is a deubiquitinating enzyme (DUB), which appears to have multiple roles in development. It has also been suggested to stabilize RNF168, facilitating the cellular response to ionizing radiation [79]. Whether USP34 plays a role in the synthesis of viral DNA and/or DNA repair at VRCs requires further investigation.

As many USPs contain the xWxxxPx motif and we have confirmed the binding of at least four such proteins to HAdV-C5 E1B-55K, we have contemplated the potential influence, whether beneficial or detrimental, of these proteins on viral infection. To address this, we examined the effect of the DUB inhibitor PR619 on viral infection. Overall, there was little difference between the time courses in the presence or absence of the drug. Although this was a relatively superficial investigation, it points to a further set of HAdV-C5 E1B-55K interactions that have a minor role in adenovirus replication. It is interesting to note that several USPs have been implicated in viral infections. For example, USP15 participates in hepatitis C virus propagation through the regulation of viral RNA translation [80], whereas USP14 inhibition prevents alphaherpesvirus infection [81]. It is possible that the use of more specific USP inhibitors would demonstrate an effect on adenovirus replication.

Large numbers of cellular proteins have been shown to interact with HAdV-C5 E1B-55K, and many more are ubiquitinated by an HAdV-C5 E1B-55K/E4orf6-dependent mechanism [27,62]. However, a role for most of these associations has not been established. We suggest that many of these interactions with proteins containing the xWxxxPx motif are fortuitous and may not be of biological significance. This does not, of course, mean that none are required for viral replication. The observation that E1B-55K proteins from species other than HAdV-C5 and HAdV-A12 do not bind to the motif supports the idea that this interaction is not essential, although it must be borne in mind that the relationship of different adenovirus species with DDR proteins, such as p53 and MRE11, varies appreciably [8,12]. Notably, the degradation of p53 and MRE11 does not occur during infection with species B, D, E, and F viruses, although a marked reduction in the level of DNA ligase IV has been observed with all species examined [8,12]. The E1B-55K protein from most adenovirus species has previously been reported to interact with p53, MRE11, and DNA ligase IV as well as other proteins, suggesting that the xWxxxPx site is not important in these cases [67].

The binding sites on HAdV-C5 E1B-55K have not been closely mapped although several point mutations in the central structured region of the protein reduce or negate binding to p53. These include point mutations or insertions at residues 180, 240, 260, 262, 309, 326, 361, and 380 (summarized in [73]). Using a different approach, it has been suggested that a binding site for p53 was located between amino acids 216 and 235 [44]. If this latter region is, indeed, the primary site of interaction, it is feasible that mutations at an appreciable distance from it must have an impact on the E1B-55K structure sufficiently to disrupt binding. Furthermore, evidence is not available to establish whether other xWxxxPx motif-containing proteins have similar binding sites. However, studies with E1B-55K mutant proteins R240A, H354in, and H373A have established a separation of function in that R240A does not bind or degrade p53 but does bind to the MRN complex or cause its degradation or the degradation of DNA ligase IV. H354in and H373A fail to interact with the MRN complex or cause its degradation but do bind to p53, causing its degradation and that of DNA ligase IV [41,82,83,84]. Such observations are consistent with the p53 and DNA ligase IV sites of interaction being in the same region of E1B-55K and the MRE11 binding site being elsewhere. Several C-terminal HAdV-C5 E1B-55K mutants have been also characterized, and the differential binding and degradation of p53, MRN, and DNA ligase IV have been demonstrated [83]. These amino acids are not part of the ‘structured core’ of the proteins yet affect protein binding. Furthermore, the separation of activity against all three substrates was observed [83]. Simplistically, however, we suggest that there is a common binding site for xWxxxPx-containing proteins and alternative binding sites for others; however, further investigation would be required to establish this.

## Figures and Tables

**Table 1:** Ad5 E1B-55K binding proteins.

Group ^1^	Protein	Amino Acid Sequence	Residues (WxxxP)	Reference
I	CEP170	NSRWRRFPTDYA	1230–1234	[62]
I	CNOT3	EAAWHHMPHPSD	622–626	[19]
I	Cullin 5	LFAWNQRPREKI	506–510	[62]
I	hnRNPR	QQNWGSQPIAQQ	597–601	[62]
I	Integrin α3 (ITGA3)	NGKWLLYPTEIT NGSWPCRPPGDL HCVWLECPIPDA	829–833 843–847 913–917	[26]
I	mSin3a	NDTWVSFPSWSE	585–589	[63]
I	MYCB2	AGKWVELPITKS VPYWNAKPAPMP DVIWRFRPNTRE	571–575 1034–1038 1138–1142	[62]
I	SQSTM1 (p62)	WPGWEMGPPGNW PGNWSPRPPRAG	198–202 206–210	[62]
I	SRSF3	PPSWGRRPRDDY	96–100	[62]
I	UBR5	LYWWGVVPFSQR DPDWLDLPPISS PPSWVPDPPAMD	494–498 835–839 1027–1031	[62]
I	USP8	IEIWKLPPVLLV	1001–1005	[62]
I	USP15	LDLWSLPPVLVV	825–829	[62]
I	USP34	IRIWLHIPAVMQ PYKWDYWPHEDV	255–259 1879–1883	[62]
I	ZNF638	GSRWDDEPHISA	104–108	[62]
II	BLM	GSLWRYRPDSLD	428–432	[21]
II	DNA ligase IV	RYSWDCSPLSMF	805–809	[18]
II	NBS1 (NBN)	MWKLLPAAGP	2–6	[22]
II	p53	SDLWKLLPENNV	23–27	[55]
II	Tab182 (TNKS1BP1)	PPSWRPQPDGEA	1507–1511	[19]
II	TIP60	LKPWYFSPYPQE	245–249	[64]
II	TOPBP1	NLQWPSCPTQYS	163–1167	[14]
III	AAV Rep52	NTIWLFGPATTG	108–112	[65]
III	AAV Rep68	EKEWELPPDSDM NTIWLFGPATTG	35–39 331–335	[66]
III	AAV Capsid	YKNWFPGPMGRT GPIWAKIPETGA	464–468 608–612	[65]
IV	TNFRSF10A	TQQWEHSPLGEL	123–127	[27]
IV	RP2	ELNWSLLPEDAV	186–190	[27]
IV	CLPTM1	YLSWILFPLLGC	481–485	[27]
IV	PDGKRB	IMLWQKKPRYEI	556–560	[27]
IV	FAS	LGIWTLLPLVLT	4–8	[27]
IV	CXADR	DIEWLISPADNQ	57–61	[27]
IV	EPHA7	DIEWLISPADNQ ELEWISSPPNGW	57–61 397–401	[27]
IV	STK11IP	HGSWSLSPPPER	774–778	[27]
IV	TRPC4AP	WGGWGGRPRPGN	25–29	[27]
IV	CLCC1	NPIWLVPPTKAL	242–246	[27]
IV	BABAM1	PKSWQVPPPAPE	73–77	[27]
V	HAX1	DDVWPMDPHPRT	173–177	[27]
V	SCAMP3	QNNWPPLPSFCP	135–139	[27]
V	SPTLC1	IEEWQPEPLVPP	64–68	[27]
V	EGFR	EGCWGPEPRDCV	471–475	[27]
V	RPN2	ASTWALTPTHYL	21–25	[27]
V	ITGB4	GSFWWLIPLLLL	712–716	[27]
V	TARS	AESWKTTPYQIA PRSWRELPLRLA	98–102 423–427	[27]
V	CANX	PEDWDERPKIPD	341–345	[27]
V	EPHA2	ELGWLTHPYGKG SVSWSIPPPQQS	42–46 456–460	[27]
V	EPHB4	DLKWVTFPQVDG	32–36	[27]
V	RPL15	DTQWITKPVHKH	147–151	[27]
V	GTF2I	SPSWYGIPRLEK	518–522	[27]
		SPTWFGIPRLER	623–627	
V	CDK6	VTLWYRAPEVLL	184–188	[27]
V	hnRNPU	GQFWGQKPWSQH	811–815	[27]
V	SLC7A5	GVWWKNKPKWLL	478–482	[27]
V	TAP1	LLHWGSHPTAFV	193–197	[27]
V	DYNC1H1	KMVWRINPAHRK TPSWLGLPNNAE	450–454 4320–4324	[27]
VI	ATR	HQLWRRFPEHVR	1443–1447	This work
VI	CHK1	KDRWYNKPLKKG LSLWDTSPSYID	264–268 328–332	This work
VI	CSB (ERCC6)	EGIWKLKPEYC	1486–1490	This work
VI	DNMT1	GSDWRDLPNIEV	1436–1440	This work
VI	LETM1	LGCWALRPECLR	73–77	This work
VI	SQSTM1 (p62)	WPGWEMGPPGNW PGNWSPRPPRAG	198–202 206–210	[62]
VI	TNKS1	RDNWNYTPLHEA MDLWQFTPLHEA ADLWKFTPLHEA TDKWAFTPLHEA	281–285 434–438 748–752 902–906	This work
VI	TNKS2	RDNWNYTPLHEA MDLWQFTPLHEA ADLWKFTPLHEA TDKWAFTPLHEA	123–127 276–280 591–595 744–748	This work
VI	USP9	KLAWDFSPEQLD GQLWLCAPQAKQ LTEWEYLPPVGP	458–462 671–675 1542–1546	This work
VI	USP15	LDLWSLPPVLVV	825–829	[62]
VI	USP33	VPSWFWGPVVTL	561–565	This work
VI	USP34	IRIWLHIPAVMQ PYKWDYWPHEDV	255–259 1879–1883	[62]
VI	XPF	RILWCPSPHATA	802–806	This work

^1^ Table listing HAdV-C5 E1B-55K binding proteins containing the xWxxxPx motif. (I) and (II), previously identified mammalian HAdV-C5 E1B-55K binding proteins with those proteins directly linked to the DDR listed in (II); (III), adenovirus-associated virus proteins previously shown to bind to HAdV-C5 E1B-55K; (IV), proteins previously shown to be ubiquitinated by HAdV-C5 E1B-55K/E4orf6 and to be degraded; (V), proteins shown to be ubiquitinated by HAdV-C5 E1B-55K/E4orf6 and not to be degraded; (VI), novel HAdV-C5 E1B-55K binding proteins identified in the present study or previously identified and examined in more detail in this study.

## Data Availability

The authors confirm that the data supporting the findings of this study are available within the article and its Supplementary Materials.

## Supplementary Materials

The following supporting information can be downloaded at: https://www.mdpi.com/article/10.3390/v15122356/s1; Figure S1: Confirmation of the Interaction of HAdV-C5 E1B-55K with Novel Cellular Binding Partners; Figures S2–S4: Localization of Selected HAdV-C5 E1B-55K Binding Proteins during Infection.
