# Supplementary material for: Identification of Adenovirus E1B-55K Interaction Partners through a Common Binding Motif
Source: Viruses. 2023 Nov 30;15(12):2356. doi: 10.3390/v15122356 (PMC10747525; doi:10.3390/v15122356)
Supplement: Supplementary file 1 [file viruses-15-02356-s001.zip › viruses-2735195-supplementary.pdf]

Supplementary Figures

Identification of Adenovirus E1B-55K Interaction Partners through a Common Binding Motif

Chalabi Hagkarim and Ip *et al*.

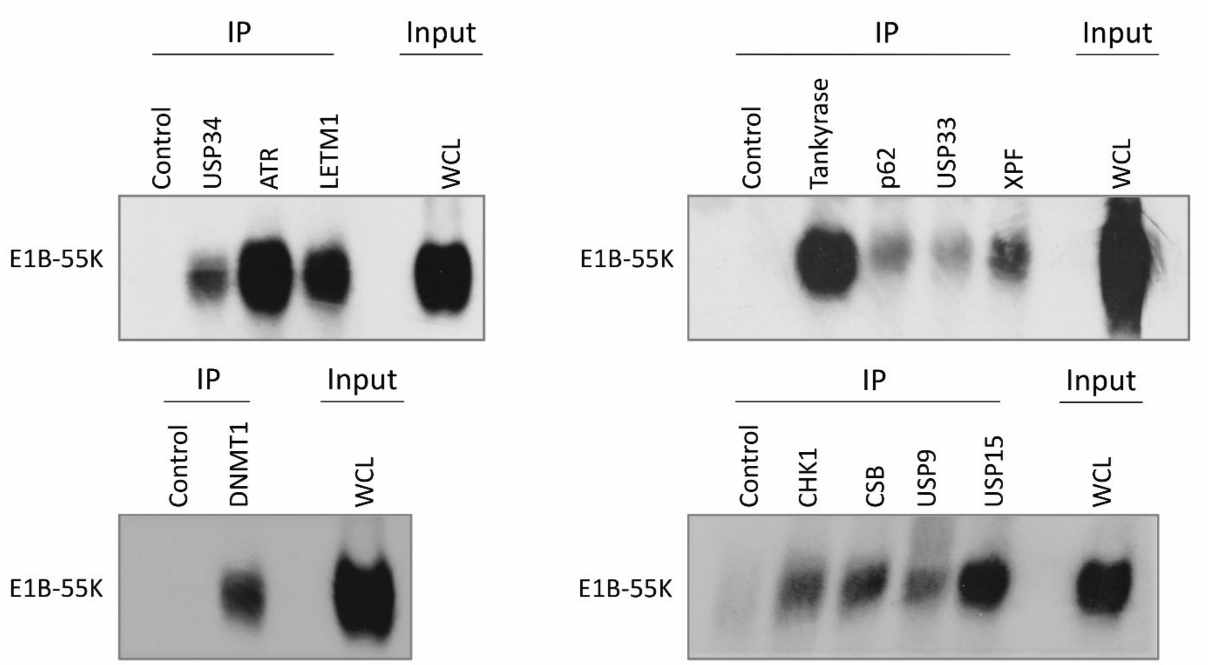

Figure S1: Confirmation of the Interaction of HAdV-C5 E1B-55K with Novel Cellular Binding Partners.

Lysates from HEK293 cells were immunoprecipitated with the antibodies shown. After immunoblotting, interacting HAdV-C5 E1B-55K proteins were detected. “Control” is an irrelevant antibody, included as a negative control, raised against either collagen type IV (rabbit) or vimentin (mouse). WCL = whole cell lysate.

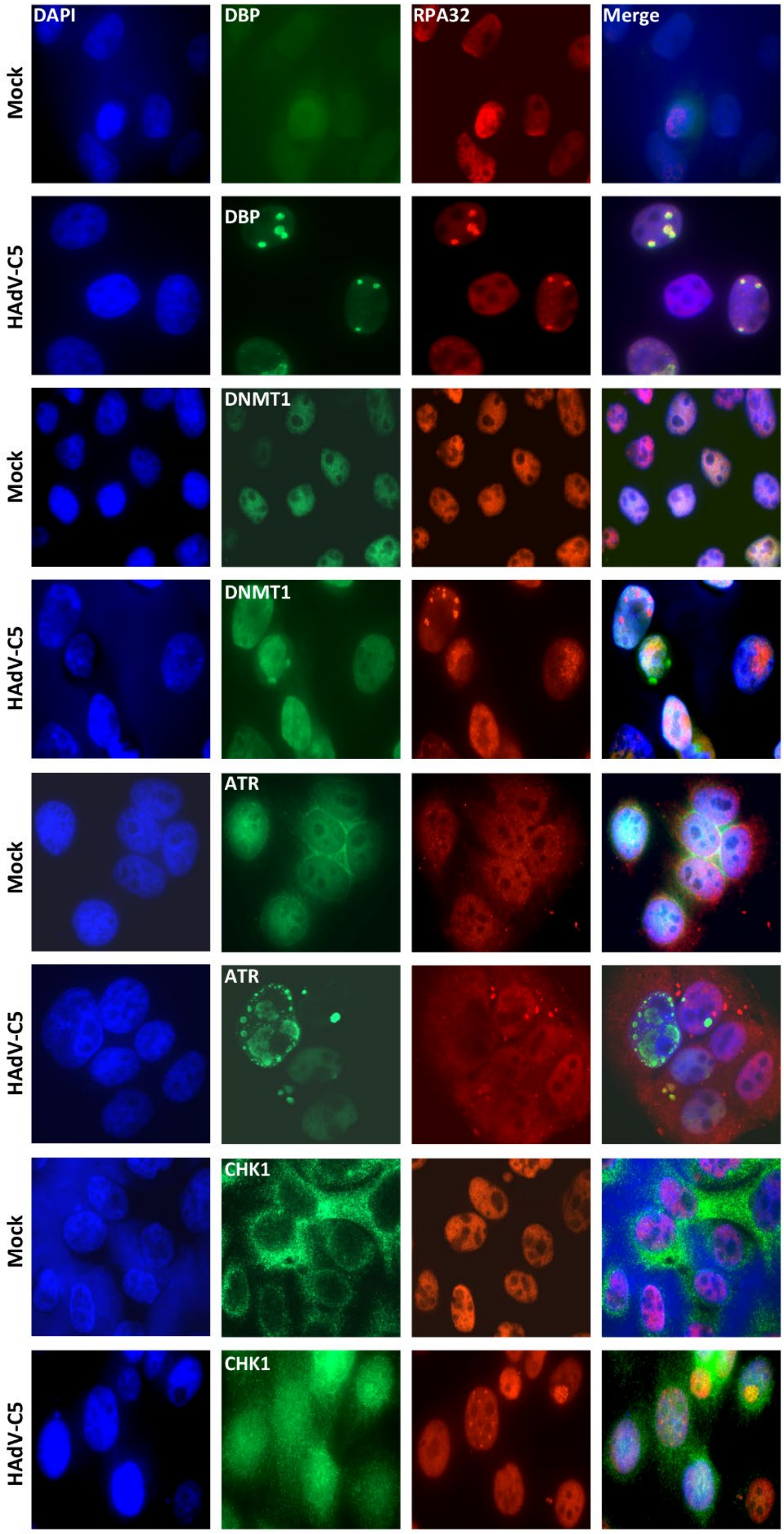

Figure S2: Localization of Selected HAdV-C5 E1B-55K Binding Proteins during Infection.

HeLa cells were mock infected or infected with HAdV-C5 (5 pfu/cell) for 24 hours. Cells were fixed as described in the Materials and Methods section before staining with the antibodies shown. VRCs were visualized by RPA32-staining. The nuclei were stained with DAPI. Images were taken with a Nikon E600 Eclipse 333 microscope (60× magnification).

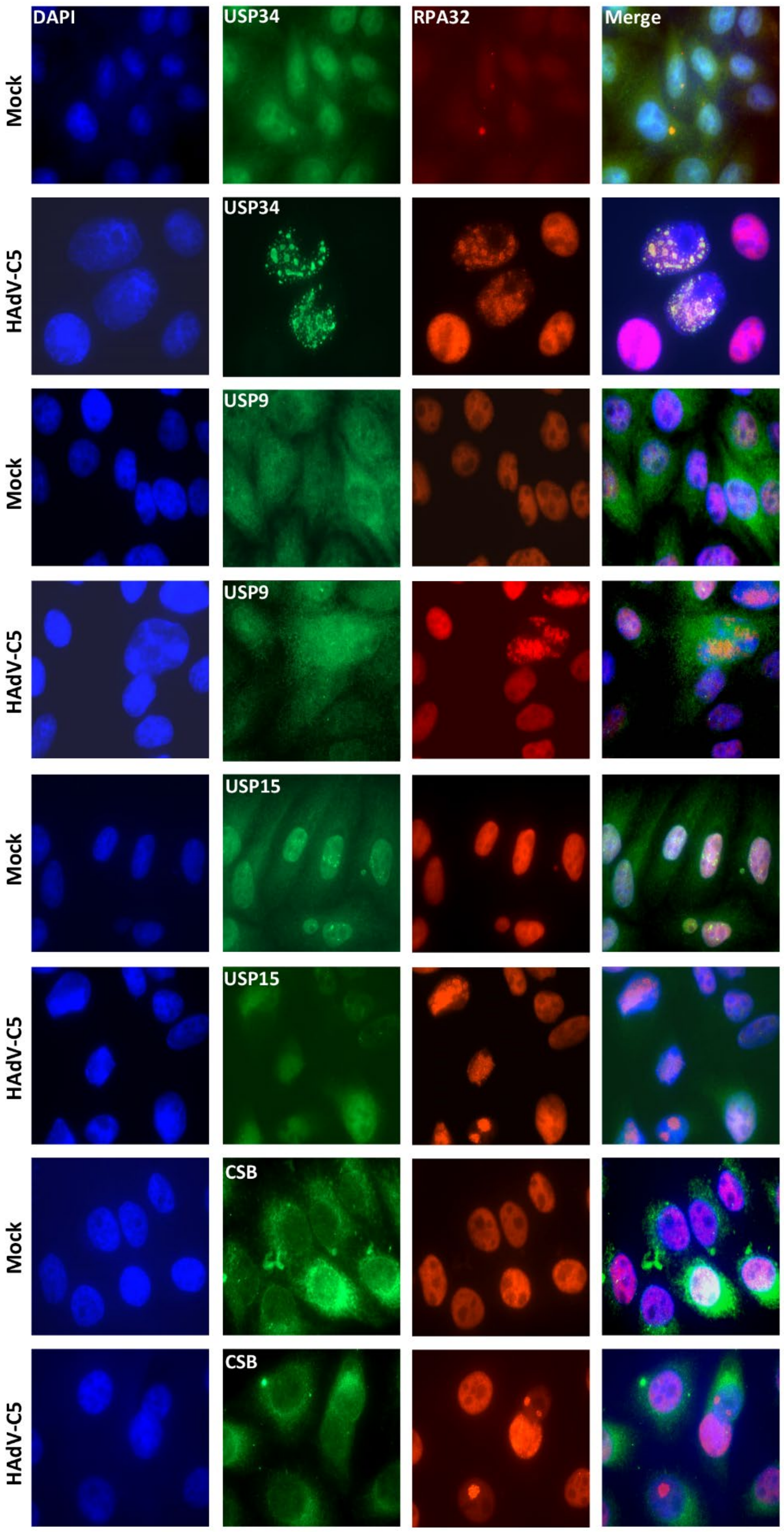

Figure S3: Localization of Selected HAdV-C5 E1B-55K Binding Proteins during Infection.

HeLa cells were mock infected or infected with HAdV-C5 (5 pfu/cell) for 24 hours. Cells were fixed as described in the Materials and Methods section before staining with the antibodies shown. VRCs were visualized by RPA32-staining. The nuclei were stained with DAPI. Images were taken with a Nikon E600 Eclipse 333 microscope (60× magnification).

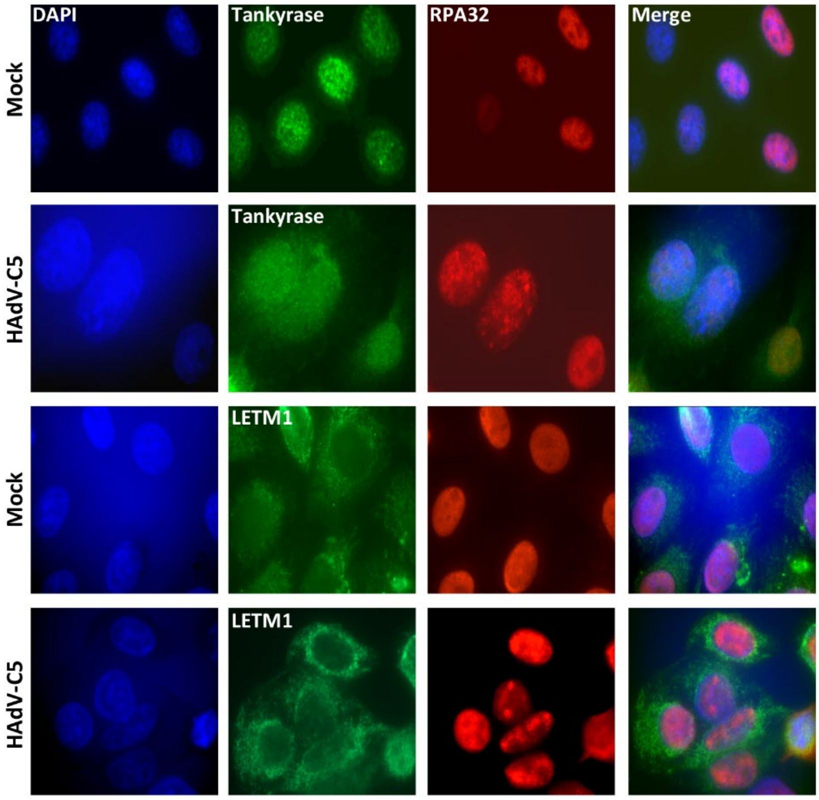

Figure S4: Localization of Selected HAdV-C5 E1B-55K Binding Proteins during Infection.

HeLa cells were mock infected or infected with HAdV-C5 (5 pfu/cell) for 24 hours. Cells were fixed as described in the Materials and Methods section before staining with the antibodies shown. VRCs were visualized by RPA32-staining. The nuclei were stained with DAPI. Images were taken with a Nikon E600 Eclipse 333 microscope (60× magnification).
